# Selectively Enhanced Development of Working Memory in Musically Trained Children and Adolescents

**DOI:** 10.3389/fnint.2019.00062

**Published:** 2019-11-06

**Authors:** Katri Annukka Saarikivi, Minna Huotilainen, Mari Tervaniemi, Vesa Putkinen

**Affiliations:** ^1^Cognitive Brain Research Unit, University of Helsinki, Helsinki, Finland; ^2^Department of Psychology and Logopedics, Medicum, Faculty of Medicine, University of Helsinki, Helsinki, Finland; ^3^Faculty of Educational Sciences, University of Helsinki, Helsinki, Finland; ^4^CICERO Learning, Faculty of Educational Sciences, University of Helsinki, Helsinki, Finland; ^5^Turku PET Centre, Turku, Finland

**Keywords:** musical training, longitudinal, working memory, updating, maintenance, development, trail-making test, Digit Span

## Abstract

In the current longitudinal study, we investigated the development of working memory in musically trained and nontrained children and adolescents, aged 9–20. We measured working memory with the Digit Span (DS) forwards and backwards tests (*N* = 106) and the Trail-Making A and B (TMT-A and B; *N* = 104) tests three times, in 2011, 2013, and 2016. We expected that musically trained participants would outperform peers with no musical training. Indeed, we found that the younger musically trained participants, in particular, outperformed their nontrained peers in the TMT-A, TMT-B and DS forwards tests. These tests all primarily require active maintenance of a rule in memory or immediate recall. In contrast, we found no group differences in the backwards test that requires manipulation and updating of information in working memory. These results suggest that musical training is more strongly associated with heightened working memory capacity and maintenance than enhanced working memory updating, especially in late childhood and early adolescence.

## Introduction

Musically trained individuals have been reported to outperform musically nontrained peers in various kinds of cognitive tests not directly related to music-making, including ones measuring long-term verbal and visual memory (Chan et al., 1998; Ho et al., 2003), executive functions (Bialystok and Depape, 2009; Degé et al., 2011; Moreno et al., 2011; Zuk et al., 2014; Saarikivi et al., 2016; however, see Schellenberg, 2011), and even intelligence (Schellenberg, 2004, 2006; Moreno et al., 2011). Executive functions (Stuss and Alexander, 2000; Jurado and Rosselli, 2007; Diamond, 2013) are cognitive processes typically divided into three related components: working memory, inhibition, and cognitive flexibility (Miyake et al., 2000; Lehto et al., 2003; Miyake and Friedman, 2012; Diamond, 2013). These three processes allow individuals to acquire, maintain, manipulate, and update representations of information of the environment, and monitor, direct and alter behavior according to these representations. Multicomponent models of working memory propose subprocesses for maintaining representations of information in memory and for manipulating this information. For instance, the influential model of Baddeley and Hitch (1974) divided working memory into two components for storage and manipulation of verbal and visual material, and a cognitive control unit (for other models, see e.g., Cowan, 1988, 1999; Unsworth and Engle, 2007). Neuroimaging and lesion studies have found separate neural functions for memory representations and attention processes that govern manipulation of this information, supporting these modular views of working memory (Postle et al., 1999; Gerton et al., 2004; Owen et al., 2005; reviews: D’Esposito et al., 1995; Miller and Cohen, 2001; Linden, 2007; Nee et al., 2012; Rottschy et al., 2012; Eriksson et al., 2015; for a discussion on differences between short-term memory and working memory, see e.g., Unsworth and Engle, 2007; Cowan, 2008; Aben et al., 2012). Multicomponent models of working memory have been validated in child studies (Gathercole et al., 2004; Gray et al., 2017), and separate brain mechanisms for encoding, maintenance and retrieval of verbal information have also been found in neuroimaging studies of children, from the age 6 onwards, and in adolescents (Gathercole et al., 2004; Siffredi et al., 2017).

Working memory and other executive functions develop from early childhood until adolescence (Cepeda et al., 2001; De Luca et al., 2003; Vuontela et al., 2003; Luna et al., 2004; Zelazo et al., 2004; Huizinga et al., 2006), following the maturation of prefrontal areas (Casey et al., 2000; Fuster, 2002; Kwon et al., 2002; Bunge and Wright, 2007; Kharitonova et al., 2013). Different executive functions however mature at slightly different rates. Development of shifting ability, which is related to cognitive flexibility, has been found to continue until adolescence (Huizinga et al., 2006; Best and Miller, 2010; Huizinga and van der Molen, 2011), and development of working memory even further, until early adulthood (Kwon et al., 2002; Huizinga et al., 2006; Satterthwaite et al., 2013).

Several cross-sectional studies have reported varying musician advantage in working memory tasks. For example, in a study by George and Coch (2011), years of musical training correlated positively with scores in both verbal and visual span tests for memory in college-aged individuals. Similarly, in another study (Talamini et al., 2016), musically trained adults outperformed nontrained peers in auditory as well as visual span tests for working memory. Finally, Zuk et al. (2014) found better performance in the Digit Span backwards test in adult musicians compared to nonmusicians, but not in musically trained children compared to nontrained peers.

Longitudinal studies with children suggest that the putative musician advantage in memory tasks may be caused by training and does not solely reflect pre-existing differences (for a discussion on problems of inferring causation from these kinds of studies, see Schellenberg, 2015). In the study by Ho et al. (2003), verbal long-term memory improved in children who continued musical training during a year-long follow-up, but not in those who did not. Similarly, in a study following the development of musically trained and nontrained children (Bergman Nutley et al., 2014), musical training was associated with improvement of verbal working memory as measured by the backwards Digit Span test, but also visual working memory as measured by a visuo-spatial working memory task. Another longitudinal study (Fujioka et al., 2006), comparing the development of children who undertook music lessons for 1 year to the development of children in a Control group, found significant improvement of working memory as measured by the Digit Span test only in the Music group. In another study (Roden et al., 2014), improvement of working memory was observed in preschool-aged children after 18 months of musical training, but not in an active Control group. In the study, effects were found specifically in tests measuring the phonological loop and the central executive subcomponents of working memory. The phonological loop was measured with the One Syllable Word Span Test, requiring participants to memorize and recite a sequence of words in the order they were presented, and the Nonword recall test, requiring participants to recite a nonword immediately after hearing it. The central executive was measured by complex span tasks requiring processing and storing information at the same time or requiring reversal of the order of a memorized sequence of information units. Last, in a recent study with quasi-random assignment of children into musical training and Control groups (Guo et al., 2018), it was found that 6 weeks of musical training improved working memory. Auditory working memory was assessed with the Digit Span forward and backward tests and with the Letter-Number Sequencing test. Both require working memory maintenance of aurally acquired information and updating and manipulation of that information in memory.

The notion that musical training might influence memory skills is further supported by findings of training-related changes in brain structures important for working memory. In their seminal study on structural differences, Gaser and Schlaug (2003) found that musicians had greater gray matter density in areas important for motor and auditory processes, and also a region of the cerebellum connected to working memory (Stoodley et al., 2012). Similarly, in a study by James et al. (2014), musical training correlated positively with gray matter density in a cerebellar areas and basal ganglia important for working memory. Another MRI study found increased thickness of frontal areas related to working memory in musicians, when compared to non-musicians (Bermudez et al., 2008).

Musical training has also been connected to changes in brain functions related to working memory. In the study by George and Coch (2011), musicians had shorter latencies of electrical brain responses (P3) to changes in visual as well as auditory stimuli, as well as larger P3 amplitudes to tonal changes. The P3 response is thought to reflect updating of working memory. In a recent study (Cheung et al., 2017), musically trained individuals outperformed nontrained peers in tasks for verbal memory and also differed in electrical brain activity measured during a verbal memory task. Specifically, musically trained individuals showed more intrahemispheric coherence in the theta band. In an fMRI study (Pallesen et al., 2010), musicians showed greater activation of brain networks for attention and working memory, including frontal, parietal, and subcortical areas than nonmusicians. In another study (Schulze et al., 2011), musicians showed different patterns of activation of brain areas during memory encoding and rehearsal of structured and unstructured tonal sequences. Nonmusicians did not show differences in activation patterns during these tasks. Musicians also outperformed nonmusicians in learning the tonal sequences.

A recent meta-analysis on cross-sectional and longitudinal studies on music-related enhancement of working memory in children and adults (Talamini et al., 2017) concluded that musicians and musically trained individuals have a clear advantage across different memory tasks when compared to nontrained peers. However, according to the results, the effect size depended on the type of information that was processed and on the memory processes required by the task. In general, the musician advantage was stronger for working memory than long-term memory, and for auditory rather than visual stimuli.

Another recent meta-analysis (Sala and Gobet, 2017a) found only a weak musician advantage in memory tasks. In this meta-analysis, however, no distinction was made between working memory and long-term memory, which may have obscured the effects of musical training on working memory reported in several cross-sectional and longitudinal studies.

In sum, there is evidence of an association between musical training and specifically verbal working memory. However, longitudinal studies have focused on school- or preschool-aged children even though executive functions are known to develop long into adolescence. As a result, it is still unclear how musical training specifically augments the development of working memory, and for how long into adulthood the possible advantage persists.

In this longitudinal study, we compared the working memory skills of 114 musically trained and nontrained children and adolescents aged 9–20. During this age range, executive functions including working memory undergo significant development, owing to the protracted development of brain areas such as the frontal lobes that important for these skills, but also begin to reach maturity (Taylor et al., 2013, 2015) The sample allows for investigating the effects of musical training on working memory, and the persistence of these effects during a developmentally highly interesting window of time. The study aims at answering questions that remain unresolved in research examining the effects musical training may have on cognitive development: does musical training augment the development of working memory, does musical training produce an advantage in working memory tasks, does this advantage persist into adulthood?

To investigate working memory, we employed two broadly studied and well-established tests: the Digit Span backward and forwards tests and the Trail-Making Test A and B. Data on performance in Digit Span tests were collected during a 3-year follow-up and the TMT-A and B tests during a 2 year follow-up. Based on previous literature, we expected musically trained children and adolescents to perform better than nontrained peers in all tests.

## Materials and Methods

### Participants

Altogether 106 children and adolescents aged 9–20 years participated in the study (Tables s 1, 2). The musically trained participants (*N* = 54, 32 females) had started training on a musical instrument approximately at age 7. They had attended or were currently attending a public elementary school that emphasizes music in the curriculum. In addition to weekly classes in classical instrumental training, school days contained music lessons such as choir and ensemble training and performances. Thus, at age 9, participants had a total of approximately 2 years of musical training and participation in the musical curriculum, at age 11, 4 years and so on. The nontrained participants (*N* = 52, 26 females) had no formal training on a musical instrument. They attended or had attended a standard elementary school with weekly group-based music lessons until the age of 13, but no instrumental tuition. No children reported hearing deficits or neurological impairments. The Music and Control groups were matched in SES and IQ (Putkinen et al., 2014).

**Table 1:** Ages of participants in the Music and Control groups per measurement year for the Digit Span Test.

Group	Year	Mean age	Minimum age	Maximum age	Standard deviation
Control	2011	12.77	8.75	15.92	2.26
Control	2013	14.61	10.43	17.81	2.45
Control	2016	17.25	13.56	20.78	2.51
Music	2011	12.46	8.92	15.83	2.31
Music	2013	14.09	10.60	17.44	2.20
Music	2016	16.76	13.50	20.29	2.30

**Table 2:** Ages of participants in the Music and Control groups per measurement year for the Trail-Making Test.

Group	Year	Mean age	Minimum age	Maximum age	Standard deviation
Control	2011	12.77	8.75	15.92	2.26
Control	2013	14.61	10.43	17.81	2.45
Music	2011	12.46	8.92	15.83	2.31
Music	2013	14.09	10.60	17.44	2.20

Written informed consent for participation was obtained from guardians of underaged participants or from over 18-year old participants themselves before the experiment. All participants also gave verbal consent for their participation. Participants were rewarded three movie tickets for taking part in the measurement. The experiment protocol was approved by the Ethical Committees of the Department of Psychology and of the Faculty of Behavioural Sciences, both at the University of Helsinki, Finland.

### Working Memory Tests

The Digit Span forwards and backwards (DS forwards, DS backwards) tests (WISC-IV, Wechsler, 2010) as well as the TMT-A and B (TMT-A, TMT-B; Poutiainen et al., 2010) were used to measure verbal working memory. In the Digit Span forwards test, subjects are aurally presented with a series of digits, and immediately recite them from memory. In the DS backwards test, participants are required to recite the presented digits in reverse order. There are multiple presentation rounds, with the experimenter always adding one to-be-memorized digit. The forwards test requires active maintenance of information in mind, and the backwards tests also manipulation of this information. Performance is evaluated by the total of digits that the participant is able to correctly recite.

The TMT-A requires the participant to connect digits printed randomly on paper by drawing a line from number to number in a sequential order. The Trail-Making Test B requires participants to alternate between connecting numbers and letters printed on the paper in order (1-A-2-B-3-C…). Both tests require maintenance of the rule of the task in mind and also maintaining awareness of where one is progressing on the sequence of digits (A) and both digits and letters (B). Performance is measured by the time taken to complete the test.

### Procedure

This study is part of a longitudinal study that started in 2003 investigating the maturation of auditory processes and executive functions in children undergoing musical training and a control group. The study entailed also EEG measurements and other tests for various cognitive skills (these data are reported elsewhere). Measurements were conducted every 2 years, with a new group of 7-year-olds recruited every 2 years. The data, therefore, contains measurements from the same participants but from different years. The data reported here include measurements conducted in the years 2011, 2013, and 2016 for the DS forwards and backwards tests and from the years 2011 and 2013 for the Trail-Making A and B Tests (The TMT was not conducted in 2016). Not all children took part in every measurement. For the tests, 25 children participated in only one measurement, 43 in two, and 38 in all three measurements. For the Trail-Making A and B Tests, 43 children participated in one measurement and 61 in both.

The cognitive tests were conducted before the EEG experiments and took a maximum of 1 h altogether. Upon arrival at the laboratory, written informed consent as well as oral consent was received from the participants. After this, the participants accompanied the experimenter to a room to complete the tests. Experimenters were graduate students, trained to work with children and adolescents and to administer the tests. The space was a comfortably lit sound-proofed room, previously used as an EEG lab, converted for testing use. The experimenter and the subject were orthogonally seated at a table. After the tests were completed, the subject was escorted to the EEG lab, where the EEG cap was attached, and the subject informed more closely about the EEG experiment. EEG measurement ensued. Participants were offered bathroom breaks when needed, and cookies and juice before the EEG measurement as well as half way through it.

### Statistical Analysis

Completion times in the TMT-A and B, and span (number of correctly recited digits) in DS forwards and backwards were included separately in analyses of test performance. The effect of age and group membership on test performance was modeled with linear mixed modeling using the lmer function [Test Score ~ Age ^*^ Group + (1|Subject)] of the Lme4 package in R (Bates, 2005; Bates et al., 2007). Age was mean centered so that the significant effect of Group indicates a group difference in the test score at average age of the participants (mean ages for the DS and TMTs were 14.39 and 13.44 years, respectively). Linear mixed modeling was selected as the analysis approach since it allows a different number of data points across subjects and takes into account the correlated nature of the data within a subject. Values below the Q1–1.5 ^*^ IQR (inter-quantile-range) or above Q3 + 1.5 ^*^ IQR were classified as outlier and replaced by the lower or upper cutoff values of this range, respectively. This procedure was applied twice for the DS backwards and Trail Making A data and five times for the Trail Making B data.

## Results

**Figure 1:**
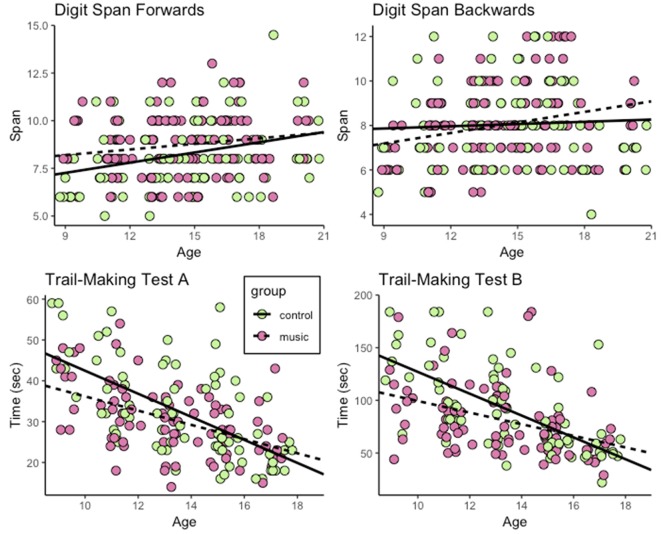
Performance of participants in the Digit Span forwards and backwards, and the Trail-Making A and B tests across all age groups. Music and Control groups represented with different colors.

**Figure 2:**
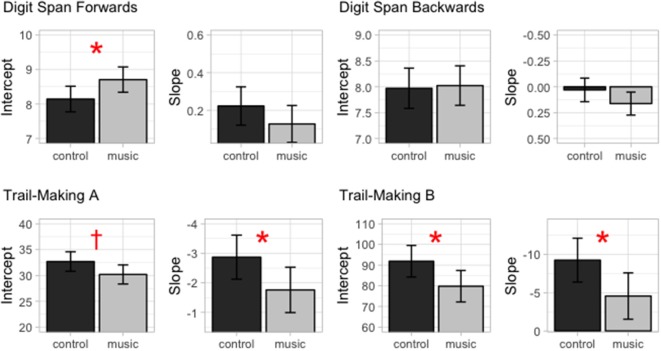
The intercepts (i.e., estimated performance at mean age) and slopes (i.e., estimated change in performance with age) for each test separately for the Music and Control groups. The errorbars indicate 95% confidence intervals. ^*^p < 0.05, ^†^*p* = 0.07.

Performance of participants in all tests except for the DS backwards test improved with age (Figures 1, 2). Musically trained participants outperformed nontrained participants in the DS forwards test, but not in the backwards test. The musically trained individuals also outperformed nontrained peers in the TMT-A. However, the group difference depended on age. The difference between performance in the Music and Control groups decreased with age. A similar age-dependent effect was also found for performance in Trail-Making Test B. The results are described in more detail below.

### Digit Span Forwards and Backwards

Performance in the DS forwards test improved with age (estimated increase in span per year: 0.22, *p* < 0.001). The Music group outperformed the Control group (estimates for the Music and Control groups: Control difference in span: 0.56, *p* < 0.05). The model revealed no evidence that this group difference was dependent on age (Group^*^Age interaction, *ns*).

For the DS backwards test, there were no significant effects of Age or Group or and no significant interaction between these predictors.

### Trail-Making A and B Tests

Subjects’ performance in the TMT-A improved with age (estimate for the decrease in completion time per year: −2.87, *p* < 0.001). There was a trend-level effect of group suggesting that the Music group outperformed the Control group in this test (estimate for the Music < Control difference: −2.50, *p* < 0.07). However, there was also a significant interaction between Age and Group indicating that the group difference was more pronounced in the younger children and decreased with age (estimate for the Music < Control differences in the change in completion time per year: 1.11, *p* < 0.05).

The performance in the Trail-Making Test B also improved with age (Estimate for the decrease in completion time per year: −9.25, *p* < 0.001). For this test, there was a significant effect of group indicating that the Music group outperformed the Control group (estimate for the Music < Control difference: −12.06, *p* < 0.05) as well as a significant interaction between Age and Group indicating that this group difference decreased with age (estimate for the Music < Control differences in the change in completion time per year: 4.69, *p* < 0.05).

## Discussion

In this study, we investigated the development of working memory in musically trained and nontrained children and adolescents. Musically trained participants outperformed nontrained peers in the DS forwards test as well as the Trail-Making A and B tests. Furthermore, the group difference in the two latter tests decreased with age. We did not find a significant difference between the Music and the Control groups in the DS backwards test.

### A Musician Advantage in the DS Forwards and Trail-Making A and B Tests

The better performance of the Music group in the DS forwards test concurs with previous research showing a musician advantage in tests for memory (Chan et al., 1998; Fujioka et al., 2006; George and Coch, 2011; Bergman Nutley et al., 2014; Roden et al., 2014; Zuk et al., 2014; Talamini et al., 2016; Guo et al., 2018; review: Talamini et al., 2017). It is noteworthy, however, that studies reporting memory enhancement in musicians have conceptual and methodological differences. In the study by Cheung et al. (2017), verbal memory enhancement was reported based on performance in a task requiring immediate as well as delayed recall of a word list, i.e., working memory as well as long-term memory. In contrast, the current study focused on working memory and employed the classical DS measure. Therefore, this study adds to the evidence for enhanced working memory in musically trained children along with earlier longitudinal studies that have used the similar span tests (Fujioka et al., 2006; Bergman Nutley et al., 2014; Guo et al., 2018).

Interestingly, we found enhancement of performance in only the forwards and not the DS backwards test. Similar results have been obtained in the study by Hansen et al. (2013) who found that musical training was associated with better performance in the DS forwards, but not backwards test. Furthermore, in their study, DS forward performance was connected to performance in musical ability tests. Along the same lines, Lee et al. (2007) found that musically trained adults outperformed nontrained peers in forwards, but not DS backwards. In their study, musically trained children, aged 12 on average, however, outperformed nontrained peer both in the forwards and DS backwards tests. Guo et al. (2018), in turn, found enhancement of the backwards but not the DS forwards after a short-term instrumental training program. Likewise, Bergman Nutley et al. (2014) found only enhancement of DS backwards performance in musically trained adults and children, but unfortunately they did not include DS forwards to allow for comparison. Thus, the literature is mixed as to whether musical training is associated with enhancement of forwards or DS backwards or both.

In any case, the current study found longitudinal evidence in a large sample of subjects in favor of selective enhancement of DS forwards in musically trained children and adolescents. Although negative results cannot be taken as evidence for the null hypothesis that there is no difference between the groups in DS backwards, the substantial statistical power of the current study indicates that a putative undetected group difference in DS backwards would have to be very small and of little practical importance.

In this study, we also found that musically trained participants outperformed nontrained peers in both of the Trail-Making Tests. Previously, adult musicians have been found to outperform nonmusicians in TMT-A and B (Bugos and Mostafa, 2011), or TMT B alone (Strong and Mast, 2019). However, for instance Bialystok and Depape (2009) and Virtala et al. (2014) found no differences between adult musicians and nonmusicians in span tests or the TMT-A or B. Our results concur with previous findings of enhanced performance in TMT-A and B in musically trained individuals but extend these findings to children and adolescents.

### Working Memory Subcomponents Measured by the Digit Span Test and the Trail-Making Test

The Digit Span test has usually been categorized as a simple span test, requiring maintenance of information in memory. Complex span tests in turn require memory maintenance of information during another, unrelated cognitive operation (Wilhelm et al., 2013). However, in a meta-analysis conducted by Redick and Lindsey (2013), the correlation between DS backward performance and performance in n-back tasks as well as a verbal complex span tests was greater than the correlation between DS forward and these tests. Because the DS backward test requires subjects to reverse the order of the strings presented in mind, it also requires working memory updating. Furthermore, the DS forward and backward tests have both been found to recruit in part separate brain networks (Manan et al., 2014). Both activate areas connected to working memory, but with the backward test more strongly activating brain areas related to cognitive control and phonological processing (Gerton et al., 2004; Yang et al., 2015).

As the DS forwards and backwards tests have been found to recruit in part separate working memory processes, it is possible that performance in one but not the other could be enhanced through training. Indeed, selective enhancement of working memory updating (Linares et al., 2018, 2019) and maintenance (Carretti et al., 2007) skills has been found as a result of working memory training in adults. Another study achieved selective impairment of working memory maintenance, but not updating with tDCS (Wang et al., 2018). Our findings of musician advantage in DS forwards, but not backwards points towards selective enhancement of working memory maintenance but not updating.

The Trail-Making Test is usually used to measure executive functions, and neuroimaging and lesion studies have identified that TMT recruits large-scale fronto-parietal brain networks related to these functions (Varjacic et al., 2018). However, there is evidence that performance in the TMT is related primarily to processing speed and working memory ability, as well as fluid intelligence (Sánchez-Cubillo et al., 2009; Satterthwaite et al., 2013). These findings are supported by evidence of genetic correlations between trail-making performance, reasoning ability and general cognitive ability, processing speed, and memory (Hagenaars et al., 2018). Research has also found differences between the cognitive processes underlying TMT-A and B performance. TMT-A is thought to rely mainly upon processing speed, and TMT-B to additionally require working memory and switching ability (Arbuthnott and Frank, 2000; Sánchez-Cubillo et al., 2009). According to a validation study of a computerized version of the TMT, TMT-B performance was explained to a large degree by inhibition and visual working memory skills (Fellows et al., 2017). Similar results were obtained in a factor analysis of TMT performance and several other neurocognitive measures in older individuals, where TMT-B performance was connected to measures of working memory and inhibition, and TMT-A to processing speed (Llinàs-Reglà et al., 2017).

There is also significant overlap between the cognitive processes that the TMT recruits. For instance, working memory skills and working memory capacity are tightly related to fluid intelligence (Kane et al., 2005; Kail, 2007; Demetriou et al., 2014; Salthouse, 2014; Heinzel et al., 2016). It has also been found that working memory predicts switching (Blackwell et al., 2009), presumably through supporting the maintenance of switching rules. Inhibitory control, in turn, may have a role in supporting working memory maintenance (Jonides et al., 1998; Zanto and Gazzaley, 2009; Getzmann et al., 2018).

In behavioral studies, DS backwards performance has been found to predict TMT-B performance, suggesting a partial overlap between the cognitive requirements of these tasks (Sánchez-Cubillo et al., 2009). Both DS backwards and TMT-B engage cognitive control more than DS forwards and TMT-A, but there are also obvious differences between test requirements. TMT-B requires switching attention from one rule and sequence of information in memory to another (letters or numbers). It also requires continuous updating of information about the respondent’s position along the series of letters or numbers they are connecting. DS backwards requires recoding a string of digits in mind into reverse order, or updating the representation of the acquired information, but does not require switching between rules or response patterns during responding. The TMT-A and B also engage specifically working memory maintenance, by requiring the participant to keep the response rule and progression along the sequence of letters and numbers in mind. As in this study, we observed a musician advantage in DS forwards but not backwards, and both the TMT-A and B, our results point towards enhancement of skills that are required by these tests but not by the DS backwards test. These include working memory maintenance for DS forwards, as well as TMT-A and B. TMT-B also requires switching ability, not required by the DS backwards test. In addition to working memory maintenance, the musician advantage in TMT-B can therefore also be explained by enhancement of switching ability.

In sum, while the Digit Span and the Trail-Making Test are routinely used to assess and connected to working memory ability, the task impurity problem complicates reaching conclusions about specifically which cognitive functions are measured and to what extent. Our results are best explained by enhancement of working memory maintenance, required by the TMT-A, B and the DS forward test. In addition, enhancement of switching ability may explain the musician advantage in TMT-B.

### How Musical Training Could Exert Selective Effects on the Development of Working Memory

Learning to play a musical instrument or sing requires working memory in a multitude of ways. For example, memorizing and producing sequences of tones when learning music by heart, and responding to changes in music when playing together with others both require working memory. It is possible that musical training during childhood could enhance working memory to the extent that this could be seen as faster development of these skills.

Augmentation of memory skills has been obtained by working memory programs (Melby-Lervåg and Hulme, 2013; Sala and Gobet, 2017b). It has been suggested that programs focusing on core working memory skills are most effective (Morrison and Chein, 2011). These programs are characterized by tasks that contain stimuli in more than one modality, require working memory maintenance and interference control, quick memory encoding and retrieval, change according to the individual’s skill level and require high engagement and focus (Morrison and Chein, 2011). Musical training matches these characteristics of core working memory training programs well. For instance, learning to play sheet music requires transformation of visual stimuli into motor actions, which produce sound stimuli. Playing from notes requires concurrent working memory maintenance and updating of visual information from notation and auditory information produced by the musician. Playing from memory adds to working memory updating and maintenance demands through requiring monitoring of the sounds and movements produced and matching them to the model of the musical piece in memory. In ensemble playing, interference control is needed in order to be able to segregate the stream of sound produced by the individuals from those produced by others. Ensemble playing also requires rapid working memory encoding and retrieval, as musicians need to follow not only their own stream of sound but also that of others, and respond to changes in others’ output. In joint improvisation, these rapid working memory encoding and retrieval requirements are accentuated. Musical training increases in challenge according to the proficiency of the individual, and successful learning and playing of music requires great engagement and focus. It is therefore feasible that musical training might influence working memory processes.

The results on selective enhancement of the participants’ working memory maintenance, but not working memory updating skills, would mean that musical training selectively engages these mechanisms and perhaps selectively supports development of one more than the other. This explanation resonates with findings of different patterns of brain activation during memory encoding and rehearsal, reflecting differences in memory processes in musicians compared to nonmusicians (Schulze et al., 2011). It is feasible that musical training might exert powerful effects specifically on working memory maintenance. Learning to play by ear relies heavily on an individual’s capability of acquiring and storing auditory information, melodies, and then reproducing this information immediately. Learning to play from notes, in turn, hones working memory maintenance in the visual domain. Conversely, classical musical training may not as much emphasize the ability to augment the presented information in mind, but rather reproduce it exactly as presented.

An alternative explanation for selective enhancement of working memory maintenance is that musical training improves selective attention. Indeed, there is evidence that selective attention underlies working memory maintenance (Sreenivasan and Jha, 2007; Berry et al., 2009; Gazzaley and Nobre, 2012). Selective attention seems to support encoding and maintenance of information in memory by shielding it from distracting information. This notion is supported by neuroimaging evidence of attenuated processing of distracting information during a working memory maintenance task (Sreenivasan and Jha, 2007). There is also tentative evidence of a musician advantage selective attention, indexed by decreased variability of frontal brain responses to attended stimuli (Strait and Kraus, 2011; Strait et al., 2015). It is possible that music training, for instance through playing in ensembles, and learning to focus on only the sound produced by one’s instrument, could develop selective attention, which is of benefit in tasks requiring memorization of aurally presented information. Selective attention may also be required and therefore trained in learning to play sheet music. For instance, in learning to play piano, there are two notations to follow—one for the right and one for the left hand. Selectively attending to this visual information is required for successful production of sound. Trail-Making Test performance has been connected to selective attention skills, as measured by the ability to recognize speech in noise (Ellis et al., 2016), but to the knowledge of the authors, similar results on a connection between specifically selective attention and Digit Span performance have not been obtained. In future studies investigating working memory in musically trained individuals, including measures for selective attention would help further elucidate this possible connection.

### Augmented Developmental Trajectories in Trained and Nontrained Participants

The difference in performance between the Music and Control group observed in this study diminished over time. It is possible that musical training enhances the development of working memory maintenance or selective attention, which can be seen as faster maturation in the Music group, but with time the Control group children attain the same level of performance. This explanation is contrasted by studies that have found enhanced working memory still in musically trained adults (Chan et al., 1998; Bialystok and Depape, 2009; George and Coch, 2011; Zuk et al., 2014; Talamini et al., 2016; Ding et al., 2018). There are, however, also contrary findings. In one study, adult nonmusicians were found to outperform musicians in tests requiring immediate as well as delayed recall of newly acquired information, with no significant group differences in performance in the TMT-A or B or DS (Virtala et al., 2014). Thus, the existence of working memory benefits associated with musical expertise in adulthood should be considered with caution.

As stated before, the task impurity problem complicates understanding of which cognitive functions are putatively most affected by musical training. Furthermore, the maturation of other executive functions may influence the maturation of subprocesses of working memory. For instance, the protracted development of inhibitory control and shifting ability influence performance in complex working memory span tasks that require these skills in addition to maintaining information in working memory (Jonides et al., 1998; Schleepen and Jonkman, 2009). Future longitudinal studies investigating working memory development should include measures that allow for disentangling the unique contributions of development in these cognitive skills to the development of working memory.

Since our study lacks baseline measurement of working memory skills prior to musical training, our results may also be explained by pre-existing differences between the two groups, instead of developmental causal explanation (for a study pointing towards pre-existing differences in intelligence, which may explain better performance in executive functions, see Schellenberg, 2015). The lack of the baseline measurement is caused by our choice to minimize the length of the experimental session when the children were only 7 years old and about to start their instrumental training. We added more behavioral and ERP paradigms gradually when the children became older and could then better cope with longer sessions. By this arrangement, we were able to minimize the number of drop-out participants—a serious problem in all longitudinal studies (for discussion, see Tervaniemi et al., 2018; Barbaroux et al., 2019).

One might consider the lack of random group allocation also as a caveat of our study. However, in our view, it is not feasible to plan a longitudinal study for several years on children and adolescents, at least if a control group is included. If the participants are not motivated, they either quit the training, do not participate in the investigations, or both. Even in shorter longitudinal studies, it has been a challenge to maintain the motivation of the participants unless the study is conducted in special circumstances such as summer camp like the study environment in the innovative study by Moreno et al. (2011). Thus, the current choice of having a longitudinal study on children who chose their music training based on their own and their family’s initiative, gives solid evidence about the development of cognitive functions of music-oriented and control children obtained in an ecologically valid context.

## Summary and Conclusions

In this study, we investigated the maturation of working memory in musically trained and nontrained children and adolescents. We found different patterns of development for different subcomponents of working memory in the trained and nontrained participants. Musically trained individuals had better performance in tests tapping working memory maintenance, but not updating, than musically nontrained individuals. However, the difference lessened over time, as nontrained participants attained a similar level of performance as trained participants. Our results extend previous findings of a musician advantage in tests for working memory by specifying which subcomponents of working memory may be most affected, and by clarifying the trajectory of enhancement from childhood into adolescence.

## Data Availability Statement

The datasets for this manuscript are not publicly available because the consent form signed by participants did not include permission for distribution of data outside the research group. Requests to access the datasets should be directed to Katri Saarikivi, katri.saarikivi@helsinki.fi.

## Ethics Statement

The studies involving human participants were reviewed and approved by The University of Helsinki Ethical Review Board in the Humanities and Social and Behavioural Sciences. Written informed consent to participate in this study was provided by the participants’ legal guardian/next of kin.

## Author Contributions

KS and VP participated in planning the experiment, conducting the measurements, analyzing the data and writing the manuscript. MT and MH were responsible for establishing the longitudinal study, planning the experiments and measurement paradigms included, and reviewing and writing the manuscript.

## Conflict of Interest

The authors declare that the research was conducted in the absence of any commercial or financial relationships that could be construed as a potential conflict of interest.
